# Early microgliosis precedes neuronal loss and behavioural impairment in mice with a frontotemporal dementia-causing CHMP2B mutation

**DOI:** 10.1093/hmg/ddx003

**Published:** 2017-01-16

**Authors:** Emma L. Clayton, Renzo Mancuso, Troels Tolstrup Nielsen, Sarah Mizielinska, Holly Holmes, Nicholas Powell, Frances Norona, Jytte Overgaard Larsen, Carmelo Milioto, Katherine M. Wilson, Mark F. Lythgoe, Sebastian Ourselin, Jörgen E. Nielsen, Peter Johannsen, Ida Holm, John Collinge, FReJA, Peter L. Oliver, Diego Gomez-Nicola, Adrian M. Isaacs

**Affiliations:** 1 Department of Neurodegenerative Disease, UCL Institute of Neurology, Queen Square, London WC1N 3BG, UK; 2 Biological Sciences, University of Southampton, Southampton General Hospital, South Laboratory and Pathology Block, Tremona Road, Southampton SO166YD, UK; 3 Department of Neurology, Danish Dementia Research Centre, Rigshospitalet, University of Copenhagen, Denmark; 4 Centre for Advanced Biomedical Imaging, Division of Medicine and Institute of Child Health, University College London, 72 Huntley Street, London WC1E 6DD, UK; 5 Faculty of Health and Medical Sciences, Department of Neuroscience and Pharmacology, Panum Institute, University of Copenhagen, DK-2200 Copenhagen N, Denmark; 6 Translational Imaging Group, Centre for Medical Image Computing (CMIC), University College London, UK; 7 Section of Neurogenetics, Department of Cellular and Molecular Medicine, University of Copenhagen, Copenhagen, Denmark; 8 Laboratory for Experimental Neuropathology, Department of Pathology, Randers Hospital, DK-8930 Randers NØ, Denmark; 9 Institute of Clinical Medicine, Aarhus University, DK-8000 Aarhus C, Denmark; 10 MRC Prion Unit, UCL Institute of Neurology, Queen Square, London WC1N 3BG, UK; 11 Frontotemporal Dementia Research in Jutland Association (FReJA), Members are listed in the Acknowledgements; 12 Department of Physiology, Anatomy and Genetics, University of Oxford, Parks Road, Oxford OX1 3PT, UK

## Abstract

Frontotemporal dementia (FTD)-causing mutations in the *CHMP2B* gene lead to the generation of mutant C-terminally truncated CHMP2B. We report that transgenic mice expressing endogenous levels of mutant CHMP2B developed late-onset brain volume loss associated with frank neuronal loss and FTD-like changes in social behaviour. These data are the first to show neurodegeneration in mice expressing mutant *CHMP2B* and indicate that our mouse model is able to recapitulate neurodegenerative changes observed in FTD. Neuroinflammation has been increasingly implicated in neurodegeneration, including FTD. Therefore, we investigated neuroinflammation in our *CHMP2B* mutant mice. We observed very early microglial proliferation that develops into a clear pro-inflammatory phenotype at late stages. Importantly, we also observed a similar inflammatory profile in *CHMP2B* patient frontal cortex. Aberrant microglial function has also been implicated in FTD caused by *GRN*, *MAPT* and *C9orf72* mutations. The presence of early microglial changes in our *CHMP2B* mutant mice indicates neuroinflammation may be a contributing factor to the neurodegeneration observed in FTD.

## Introduction 

Frontotemporal dementia (FTD) is a common cause of young-onset dementia. FTD is characterized by personality and behavioural changes, or language impairment, due to atrophy of the frontal and temporal lobes (1). In addition, FTD also shares a clinical spectrum with amyotrophic lateral sclerosis (ALS), with both diseases sharing common pathologies and genetic causes (2).

Familial FTD can be caused by mutations in a number of diverse genes. The most common causes of FTD are mutations in the genes that encode *C9orf72*, tau (*MAPT*) and progranulin (*GRN*), with less common causes identified as mutations in valosin-containing protein, TDP-43 (*TARDBP*), fused in sarcoma and charged multivesicular body protein 2B (*CHMP2B*) (3).

A mutation in CHMP2B was identified as the cause of an autosomal dominant form of FTD, termed FTD-3 (4,5), which we will refer to as *CHMP2B*-FTD. The location of the mutation in a splice acceptor site results in the production of two variants of C-terminally truncated CHMP2B proteins: CHMP2B^Intron5^ in which the final 36 amino acids are replaced with a single valine residue, or CHMP2B^Δ10^ in which the final 36 amino acids are replaced with 29 nonsense residues (5). Further evidence that C-terminal truncation of CHMP2B leads to FTD was found in a subsequent study in a Belgian FTD cohort, where a distinct truncation mutation CHMP2B^Q165X^ that leads to the loss of the final 49 amino acids was discovered (6,7).

The normal function of CHMP2B is as part of the endosomal sorting complex required for transport III (ESCRT-III), which is necessary for membrane deformation during intraluminal vesicle biogenesis in the maturation of endosomes (8). Mature endosomes, or multivesicular bodies, ultimately fuse with lysosomes to allow degradation of endosomal content (8,9). Alternatively, MVBs can also fuse with autophagosomes, to form hybrid organelles known as amphisomes, prior to lysosomal fusion (9,10). Mutant CHMP2B^Intron5^ has been shown to affect the maturation of both endosomes and autophagosomes (11–14). We recently reported in a CHMP2B mouse model of FTD that expresses human CHMP2B^Intron5^ at endogenous levels, that both neurons and microglia accumulate progressive and specific lysosomal storage inclusions (15). Similar inclusions were also identified in the frontal cortex of patients with *CHMP2B*-FTD (15). Interestingly, a lysosomal storage disorder phenotype has also been reported in patients and mouse models with *GRN* mutations, suggesting some mechanistic overlap between *GRN* and *CHMP2B* (16,17).

In addition to cell autonomous effects in neurons such as endolysosomal dysfunction, there is increasing focus on the contribution of microglia and neuroinflammation to the progression of neurodegeneration. Neuroinflammation has been implicated in the pathophysiology of neurodegenerative disease, including Alzheimer’s disease, Parkinson’s disease, ALS (18) and FTD caused by *GRN* mutations (19). Substantial evidence shows that microglial cells participate in the progression of the neurodegenerative process, and recent strategies aiming at inhibiting microglial proliferation have shown beneficial effects in mouse models of prion disease (20), AD (21–23) and ALS (24). We therefore performed a detailed characterization of the activation and proliferation of microglia in conjunction with assessment of brain volume, neuronal loss and longitudinal behavioural analyses in our CHMP2B mutant mouse model. We show that microglial activation is an early event in *CHMP2B*-FTD, which precedes neuronal loss and behavioural defects. In addition, we report that a similar microglial activation profile exists in *CHMP2B*-FTD patient brain. These data implicate microglial activation as a potential pathway for early therapeutic intervention.

## Results

### Volume loss in aged mutant CHMP2B mouse brain

**Figure 1:**
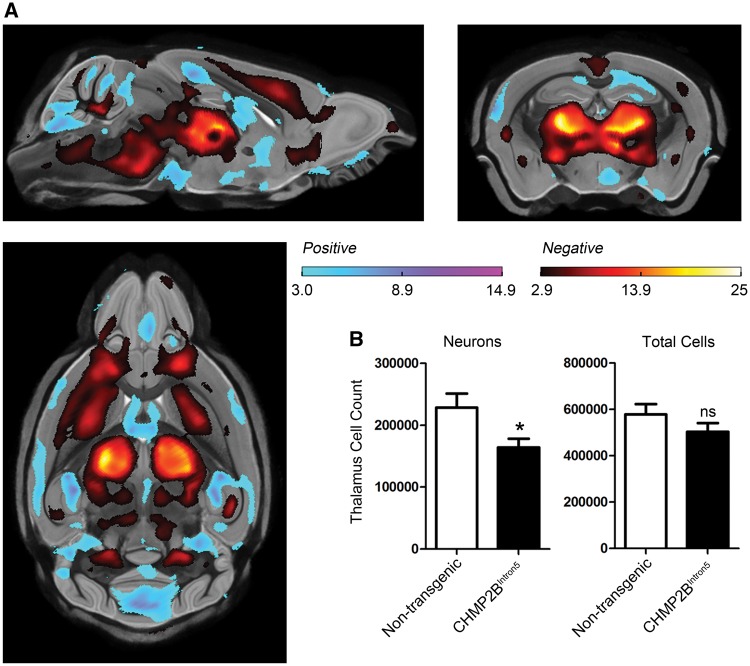
Volume loss is accompanied by neuronal loss in 18-month-old CHMP2B^Intron5^ mouse brain. **(A)** High resolution *ex vivo* MRI results, showing statistically significant FDR-corrected (q = 0.05) t-statistics overlaid on the group-wise registration average image, revealing local structural differences between the brains of the CHMP2B^Intron5^ mice (*n* = 9) relative to non-transgenic controls (*n* = 10). Regions highlighted in red represent a volume decrease in the CHMP2B^Intron5^ brains, and regions highlighted in blue represent a volume increase. Significant volume loss can be readily observed in the thalamus, cortex and brain stem of CHMP2B^Intron5^ brains. Sagittal, coronal and horizontal views are shown. **(B)** Stereological cell counts reveal a significant decrease in neuron number in 18-month-old CHMP2B^Intron5^ thalamus (*n* = 5) when compared with non-transgenic control (*n* = 5). There is no difference in the total number of cells in the thalamus. *P < 0.05, *t*-test. Data are shown as mean ± SEM.

For these studies, we utilized our CHMP2B^Intron5^ mice, which express mutant truncated human CHMP2B^Intron5^ at endogenous levels (25). CHMP2B^Intron5^ mice were compared with mice overexpressing wildtype human CHMP2B (CHMP2B^Wildtype^) (25) and non-transgenic controls. In order to investigate whether brain atrophy occurs in CHMP2B^Intron5^ mice we used high resolution *ex vivo* MRI to measure brain volume at 18 months of age. We focused on this late-stage time point to maximize the possibility of detecting changes as behavioural impairments only become apparent at 18 months of age (described below) and early time points do not show overt inclusion pathology (25). We applied a tensor-based morphometry map that does not require manual delineation of regions of interest or isolation of specific structures (26). This automated analysis revealed significant bilateral volume loss in the thalamus and cortex of CHMP2B^Intron5^ mice compared with both non-transgenic (Fig. 1A) and CHMP2B^Wildtype^ controls (Supplementary Material, Fig. S1) and no significant difference between CHMP2B^Wildtype^ and non-transgenic mice (not shown). We have shown previously that these particular brain regions harbour inclusion pathology (15,25), and these findings are consistent with brain volume changes observed by MRI in CHMP2B-FTD patients (6,27,28), suggesting that brain atrophy is occurring in CHMP2B^Intron5^ mice.

### Neuronal loss in aged mutant CHMP2B mouse brain

In order to determine whether the observed volume loss in CHMP2B^Intron5^ mice was due to loss of neurons, the brains that had undergone MRI were serially sectioned and stereological cell counts performed in the thalamus. Both the total cell number and the number of neurons were counted. The number of neurons in the thalamus of CHMP2B^Intron5^ mice was found to be significantly decreased compared with non-transgenic controls (Fig. 1B), indicating that the volume loss observed by MRI was due to loss of neurons. Interestingly, the total number of cells was not significantly altered (Fig. 1B); this is likely to be due to the gliosis that we have previously reported in the thalamus of CHMP2B^Intron5^ mice at 18 months of age (25). These data are the first to show that endogenous expression levels of mutant CHMP2B^Intron5^ can directly cause neuronal loss *in vivo* and indicate that our model is faithfully recapitulating key neurodegenerative features of *CHMP2B*-FTD.

### Social and motor deficits in aged mutant CHMP2B mice

Patients with FTD, including those with *CHMP2B* mutations, display changes in personality and behaviour as well as motor dysfunction (6,29); therefore behavioural testing was used to assess disease-relevant deficits in the CHMP2B^Intron5^ mouse model at 6, 12 and 18 months of age compared with both non-transgenic and CHMP2B^Wildtype^ controls. As this was the first behavioural characterization of these transgenic lines, a battery of tests was performed at each timepoint to facilitate interpretation of the data. First, to assess exploratory behaviour, locomotor activity was measured in an open field arena. Although there was no difference in overall activity between any of the lines at each individual timepoint (Supplementary Material, Fig. S2A), examining time-bins across a single trial demonstrated that CHMP2B^Intron5^ mice were significantly less active towards the middle of the session at 18 months of age (Supplementary Material, Fig. S2B). Analysis of the same open field data for proportion of activity in the central zone did not show any difference between genotypes (Supplementary Material, Fig. S2C); therefore, as a further measure of similar approach-avoidance anxiety-like behaviour, mice were also analysed on an elevated plus-maze, and no differences in time spent in the open arms between genotypes was observed (Supplementary Material, Fig. S2D).

Next, we assessed cognitive performance using tests of short-term memory. In the short-term exploratory spatial memory tasks – spontaneous alternation and Y-maze spatial novelty preference tests – mice of all genotypes performed well above chance levels (Supplementary Material, Fig. S2E and F) with no significant difference in their activity in the apparatus (data not shown). Together, these data suggest that CHMP2B^Intron5^ mice do not display anxiolytic phenotypes or behaviour indicative of disinhibition or profound cognitive dysfunction.

**Figure 2:**
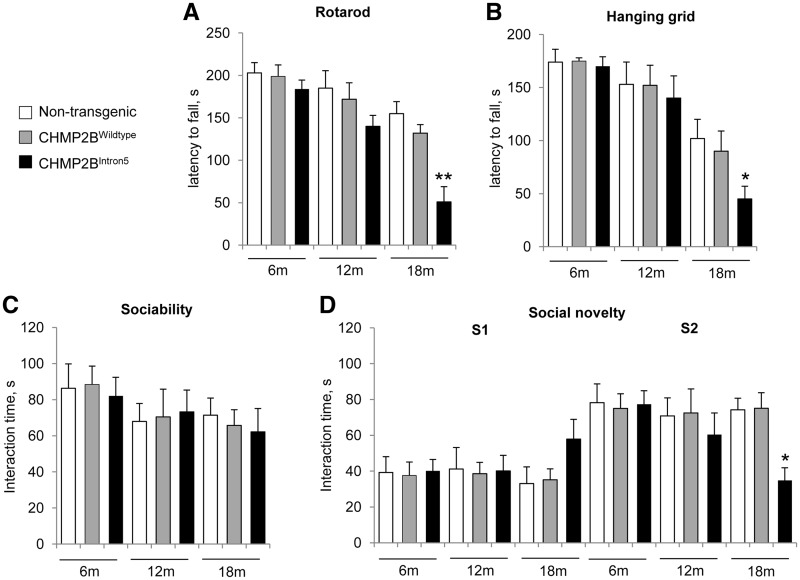
Social behaviour and motor impairments in 18-month-old CHMP2B^Intron5^ mice. Longitudinal behavioural phenotyping was performed on CHMP2B^Intron5^, CHMP2B^Wildtype^ and non-transgenic mice (*n* = 12 per group) at 6, 12 and 18 months of age. **(A**) and (**B)** Performance on the accelerating rotarod (A) and the hanging grid test (B) was significantly impaired in 18-month old CHMP2B^Intron5^ animals compared with non-transgenic and CHMP2B^Wildtype^ control mice. (**C**) and (**D**) In the social interaction test, no deficits in sociability were identified (C), whereas in the social novelty section of the task (D), CHMP2B^Intron5^ mutants spent significantly less time with the new intruder mouse (S2) at 18 months of age versus non-transgenic and CHMP2B^Wildtype^ animals, **P < 0.01, * *P* < 0.05; ANOVA with Bonferroni post-hoc test). Data are shown as mean ± SEM.

We then examined motor coordination and grip strength using the accelerating rotarod and hanging grid tests. Interestingly, 18-month-old, but not 6- or 12-month-old CHMP2B^Intron5^ mice performed significantly worse than non-transgenic and CHMP2B^Wildtype^ mice in both of these tasks (Fig. 2A and B), demonstrating that CHMP2B^Intron5^ mice develop late-onset motor defects. In addition, we assessed depression-like behaviour in the forced swim test and observed no difference between genotypes at any timepoint (Supplementary Material, Fig. S2G).

We went on to examine sociability and preference for social novelty in CHMP2B^Intron5^ mice using a modified three chamber assay. Briefly, in the habituation stage (stage 1), the test mouse was allowed to explore all three chambers of the apparatus. In stage 2 (testing for sociability), an unfamiliar stranger mouse enclosed in a wire cage was placed in either the left or right chamber, and the test mouse was allowed to freely explore all chambers. CHMP2B^Intron5^ mice spent the same amount of time interacting with the stranger mouse as the non-transgenic and CHMP2B^Wildtype^ mice at 6, 12 and 18 months of age, demonstrating that no specific defect in sociability occurs in these lines during ageing (Fig. 2C). In stage 3 of the test the preference for social novelty was analysed, with stranger 1 retained in its original position, and a novel unfamiliar mouse (stranger 2) introduced to the opposite chamber. Interestingly, at 18 months of age, but not earlier timepoints, CHMP2B^Intron5^ mice spent significantly less time interacting with stranger 2, demonstrating a decreased interest in novel social interaction (Fig. 2D). These data were corroborated by quantification of the time spent in each chamber of the apparatus (Supplementary Material, Fig. S3 A–C). This apparent lack of interest in novel social interaction was not due to defects in olfaction, as the olfactory response of the CHMP2B^Intron5^ mice to both attractive and aversive scents was normal in these animals (Supplementary Material, Fig. S3D). In summary, these data show specific FTD-like changes in social behaviour and late-onset motor deficits in CHMP2B^Intron5^ mice.

### Early and progressive neuroinflammatory reaction in mutant CHMP2B mouse brain

**Figure 3:**
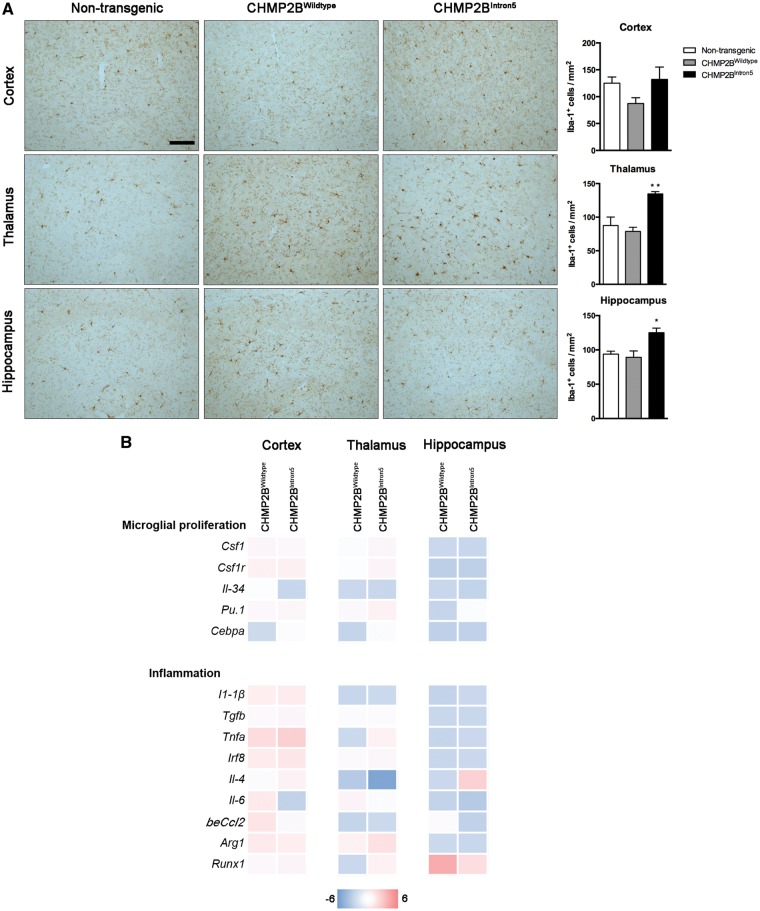
Early neuroinflammatory changes in CHMP2B^Intron5^ mouse brain. **(A)** Representative microphotographs and quantification of microglial cells (Iba-1) in cortex, thalamus and hippocampus of 3-month-old non-transgenic, CHMP2B^Wildtype^ and CHMP2B^Intron5^ mice (n = 3 per group). Scale bar: 100 µm. Data are shown as mean ± SEM. *P  <  0.05, **P  <  0.01 versus both non-transgenic and CHMP2B^Wildtype^. **(B)** Analysis of mRNA levels of microglial proliferation and inflammation associated genes in cortex, thalamus and hippocampus of 3-month-old non-transgenic, CHMP2B^Wildtype^ and CHMP2B^Intron5^ mice (*n*  =  4 per group). Data are represented with a colour code (blue to red) as fold change of CHMP2B^Wildtype^ vs. non-transgenic and CHMP2B^Intron5^ versus non-transgenic mice from −6 to 6.

Having determined that expression of CHMP2B^Intron5^ leads to neuronal loss and behavioural changes at late stages (18 months) in our mouse model, we next sought to determine whether neuroinflammation contributes to this process. We therefore looked for early microglial changes at 3 months of age. We assessed the number of microglial cells present in different brain regions of 3-month-old non-transgenic, CHMP2B^Wildtype^ and CHMP2B^Intron5^ mice. Results showed a significant increase in the number of microglia (Iba-1^+^) both in the thalamus and hippocampus of CHMP2B^Intron5^ mice compared with non-transgenic and CHMP2B^Wildtype^ controls (Fig. 3A). We then analysed the expression of well-known markers of microglial proliferation at the same time point: components of the *Csf1/Csf1r* signalling pathway, *Cebpa* and *Pu.1* (20), but we observed no significant changes in the mRNA levels of these markers when we compared CHMP2B^Wildtype^ to CHMP2B^Intron5^ mice (Fig. 3B, upper panel). The expression of inflammatory cytokines was also unaltered (Fig. 3B, lower panel).

**Figure 4:**
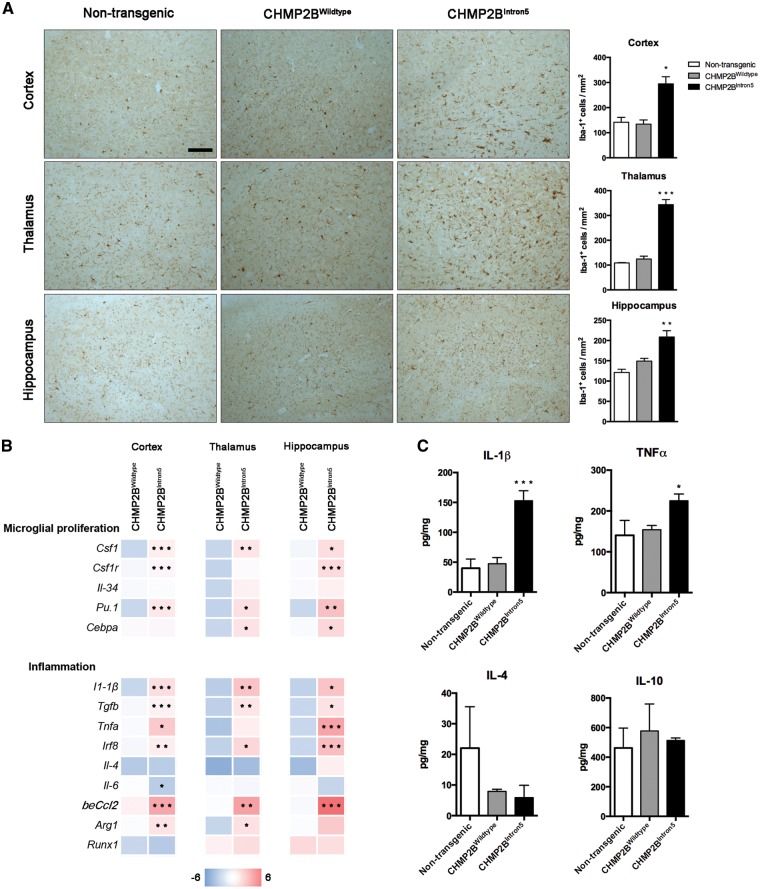
Late stage neuroinflammatory changes in CHMP2B^Intron5^ mouse brain. (**A**) Representative microphotographs and quantification of microglial cells (Iba-1) in cortex, thalamus and hippocampus of 18-month-old non-transgenic, CHMP2B^Wildtype^ and CHMP2B^Intron5^ mice (*n*  =  3 per group). Scale bar: 100 μm. Data are shown as mean ± SEM. *P  <  0.05, **P  <  0.01, ***P  <  0.001 versus non-transgenic. (**B**) Analysis of mRNA levels of microglial proliferation and inflammation associated genes in cortex, thalamus and hippocampus of 18-month-old non-transgenic, CHMP2B^Wildtype^ and CHMP2B^Intron5^ mice (*n*  =  4 per group). Data are represented with a colour code (blue to red) as fold change of CHMP2B^Wildtype^ versus non-transgenic and CHMP2B^Intron5^ versus non-transgenic from −6 to 6. *P  <  0.05, **P  <  0.01, ***P  <  0.001 versus CHMP2B^Wildtype^. (**C**) Protein analysis of cortical samples showing a significant overexpression of proinflammatory cytokines (*n*  =  4 per group). Data are shown as mean ± SEM. *P  <  0.05, ***P  <  0.001 versus both non-transgenic and CHMP2B^Wildtype^.

At late stages of the disease (18 months), there was also a significant expansion of the microglial population, with a 2- to 4-fold increase in the number of Iba-1^+ ^cells in the cortex, thalamus and hippocampus of CHMP2B^Intron5^ mice compared with non-transgenic and CHMP2B^Wildtype^ mice (Fig. 4A). Analysis of the microglial coverage of the cortex showed a 3- to 3.5-fold increase in CHMP2B^Intron5^ when compared with non-transgenic and CHMP2B^Wildtype^ mice, with no significant differences in the microglial distribution across CHMP2B^Intron5^ cortical layers (LIII-IV: 3.2  ±  0.15; LV: 3.6 ± 0.20; LVI: 3.3  ±  0.62; mean  ±  SEM). This was coupled with a significant up-regulation of specific genes known to be involved in the proliferation of these cells, including *Csf1*, *Csf1r*, *Pu.1* and *Cebpa* (20) (Fig. 4B, upper panel). Increased microglial numbers were also associated with an exacerbated inflammatory reaction in the brain of CHMP2B^Intron5^ mice, as shown by significantly high expression of the proinflammatory cytokines IL1β and TNFα both at mRNA and protein level that was consistent in all the analysed regions (Fig. 4B (lower panel) and C).

**Figure 5:**
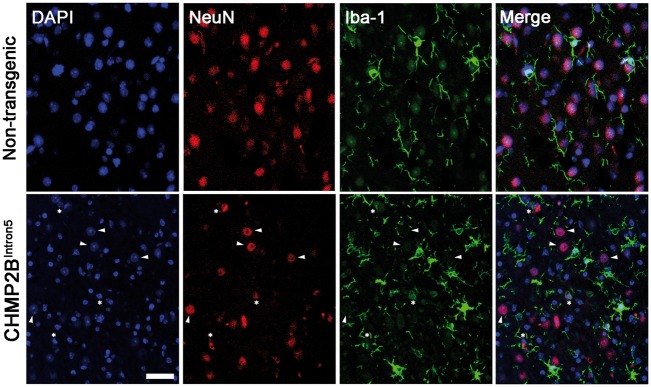
Microglial engulfment of dying neurons in aged CHMP2B^Intron5^ mouse brain. Confocal analysis of the association of microglia (Iba1^+^, green) with neurons (NeuN^+^, red) in the thalamus of 18-month-old CHMP2B^Intron5^ mice. DAPI (blue) stains nuclei and is used to identify apoptotic cells (condensed chromatin). Arrowheads indicate normal neuronal nuclei, stars indicate condensed neuronal nuclei characteristic of apoptosis, which are opposed by microglial processes. Scale bar represents 20 µm.

We observed association of apoptotic neurons (NeuN^+ ^with condensed chromatin) with activated microglial cells, which were often found in close apposition or engulfing NeuN cells (Fig. 5**)**, indicating a microglial reaction to neurodegeneration. However, we did not observe microglial phagoptosis (phagocytosis of endangered but alive neurons). These changes were not detected in CHMP2B^Wildtype^ or non-transgenic mice (Fig. 5).

Overall, these results show an early microglial reaction in CHMP2B^Intron5^ mouse brain. This progresses to an overt microgliosis characterized by a proinflammatory phenotype at late stages of the disease, which likely contributes to the neurodegenerative process.

### Spinal cord and muscle pathology in late stage CHMP2B^Intron5^ mice

**Figure 6:**
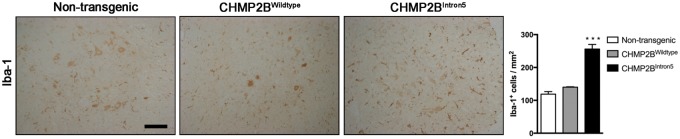
Microgliosis in 18-month-old CHMP2B^Intron5^ mouse spinal cord. Representative microphotographs and quantification of microglial cells (Iba-1) in the anterior horn of the spinal cord of 18-month-old non-transgenic, CHMP2B^Wildtype^ and CHMP2B^Intron5^ mice. Data are shown as mean ± SEM. *P  <  0.05 versus CHMP2B^Wildtype^ and non-transgenic (*n*  =  3 per group). Scale bar: 100 µm.

Given that CHMP2B^Intron5^ animals show motor deficits by the end stage of the disease (Fig. 2A and B), we further explored the alterations in the spinal cord and muscle of these mice at 18 months of age. There was a significant 2-fold increase in the number of microglial cells (Iba-1^+^) in CHMP2B^Intron5^ spinal cord when compared with CHMP2B^Wildtype^ and non-transgenic mice (Fig. 6). We also performed fibre type analysis in quadriceps muscles as changes in fibre type composition, such as an increase in type IIa fibres, can be indicative of denervation (30). CHMP2B^Intron5^ mice had more type IIa fibres than CHMP2B^Wildtype^ and non-transgenic mice (8.3  ±  3.7, 1.7  ±  1.2 and 4.2  ±  1.9, respectively, mean  ±  SEM), which reached significance only for the comparison with CHMP2B^Wildtype^ (Supplementary Material, Fig. S4), suggestive of denervation in CHMP2B^Intron5^ mice. Therefore, alterations in both the spinal cord and distal processes may contribute to the motor phenotypes we observe.

### Microglial activation in *CHMP2B*-FTD brain parallels late stage CHMP2B^Intron5^ mouse brain

**Figure 7:**
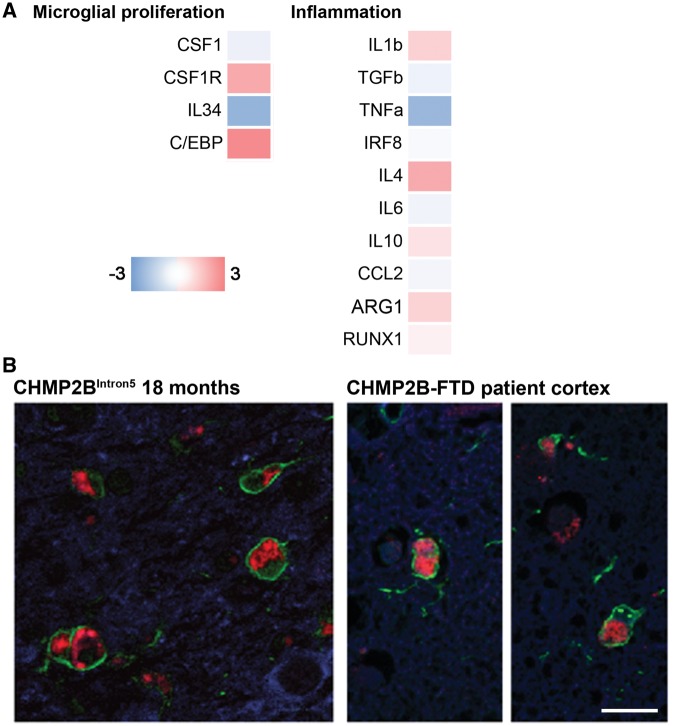
Similar activated microglia profiles in *CHMP2B* patient brain and 18-month-old CHMP2B^Intron5^ mouse brain**. (A)** Analysis of mRNA levels of microglial proliferation and inflammation associated genes in the frontal cortex of *CHMP2B*-FTD (*n*  =  4) and age-matched controls (*n*  =  5), as performed for 18-month-old CHMP2B^Intron5^ mice (Fig. 4B). Data are represented with a colour code (blue to red) as fold change of *CHMP2B*-FTD versus age-matched controls from −3 to 3. **(B)** Representative images of microglia (Iba1, green) in *CHMP2B* patient frontal cortex and CHMP2B^Intron5^ thalamus reveals activated microglia containing characteristic mutant CHMP2B lysosomal storage-like autofluorescent pathology in both patient brain and mouse brain. Autofluorescence is pseudo-coloured in red, neurons (β-III tubulin, blue) are also shown in the left-hand image and nuclei (DAPI, blue) are shown in the right-hand image). Scale bar represents 25 µm.

RT-PCR in *CHMP2B* patient frontal cortex showed a non-significant trend towards a pro-inflammatory profile similar to that in 18-month-old CHMP2B^Intron5^ mouse brain, although there was a high level of variability across the genes tested (Fig. 7A). We have previously shown that autofluorescent lysosomal storage-like pathology is a feature of the CHMP2B mutation and is increased in microglia in *CHMP2B* patient frontal cortex when compared with both neurodegenerative disease and normal controls (15). A direct comparison between mouse and human revealed activated microglia containing lysosomal storage pathology that are strikingly similar in CHMP2B^Intron5^ mouse brain and *CHMP2B* patient frontal cortex (Fig. 7B). These data suggest our CHMP2B^Intron5^ mice may provide an accurate model of microglial activation during disease pathogenesis.

## Discussion

Neurodegenerative conditions are often associated with a neuroinflammatory reaction characterized by the proliferation and activation of microglia (18). However, the exact contribution of this gliosis to the progression of neurodegeneration in the disease process is not well understood. The time-course of the onset of these pathologies is difficult to dissect in patient samples, which by necessity are only examinable at the end stage of disease. In order to better understand the temporal order of gliosis, neuronal loss and onset of behavioural symptoms in FTD, we have investigated these pathologies in detail in a mouse model of *CHMP2B*-FTD that expresses endogenous levels of mutant CHMP2B.

### Volume and neuronal loss in mutant CHMP2B mice

We report that mutant CHMP2B^Intron5^ mice have decreased brain volume, due to neuronal loss, in the cortex and thalamus at 18 months of age. These are areas that we have previously reported as showing a significant burden of neuronal and microglial autofluorescent aggregates (15). It is striking that the thalamus is the most affected brain region in our mutant CHMP2B mouse model. Mutant CHMP2B expression is driven by the hamster prion protein gene promoter, which is known to express in the thalamus (31,32), so this likely explains the thalamus pathology we observe. However, the pathology is more severe than in other models which use the same promoter. For instance, the Tg2576 transgenic mouse drives expression of mutant APP using the same hamster prion protein gene promoter (33) and there are no amyloid plaques in the thalamus even at 16 months of age, while several other brains regions have a significant plaque load (34). This suggests that the thalamus may be particularly sensitive to mutant CHMP2B, which is consistent with several imaging studies that show the thalamus is affected in FTD (35). Indeed, the thalamus is one of the earliest brain regions affected in *C9orf72* mutation carriers, up to 25 years before expected symptom onset (36). Furthermore, the thalamus is part of the salience network, which consistently shows decreased network connectivity in FTD (35). Interestingly, we also detected brain regions with increased brain volume. The reasons for this are unclear but could include compensatory mechanisms. For example, several groups have shown with MRI that the default mode network has increased connectivity in FTD and that this correlates with decreased connectivity in the salience network (35). It will therefore be interesting to investigate network connectivity in our CHMP2B mutant mouse model in future studies.

We observed microglia engulfing apoptotic neurons in the thalamus but not healthy neurons at 18 months of age. This indicates that while microglia in CHMP2B^Intron5^ mice are involved in clearance of cellular debris, neuronal loss is not directly caused by aberrant microglial phagocytosis of healthy or at risk neurons, known as phagoptosis (37). This is the first report of mutant CHMP2B^Intron5^ causing neurodegeneration *in vivo*. Therefore, we provide the first direct link between CHMP2B^Intron5^ and neuronal loss, the core feature of FTD. Importantly, the neurodegeneration is caused by endogenous expression levels of CHMP2B^Intron5^. This suggests the phenotypes we observe accurately model the neurodegenerative processes caused by mutant CHMP2B rather than being artefacts caused by over-expression of a toxic protein.

### Behavioural changes in mutant CHMP2B mice

One important feature of our CHMP2B^Intron5^ model is that the endogenous expression level of the transgene facilitates both the identification of *bona fide* presymptomatic features of CHMP2B dysfunction in addition to the longitudinal analysis of neuropathology and behaviour. Here, we report a very specific and late-onset defect in preference for social novelty but not sociability in our CHMP2B^Intron5^ mice. Interestingly, an impairment in social novelty using the same behavioural paradigm was reported in a mouse model expressing CHMP2B^Intron5^ under the control of the CamKIIa promoter to induce forebrain-specific expression at below endogenous levels (38). The identification of the same behavioural phenotype in two distinct mouse models that do not use over-expression implies this is a genuine effect of CHMP2B^Intron5^ to induce FTD-like behavioural changes. Importantly, impairments in social functioning are a key feature of FTD (39) and are prominent in FTD caused by CHMP2B mutation (29). Impairment in the social novelty paradigm is an indication of altered social functioning and has been utilized across other FTD-relevant models (40). For example, reductions in social interest and/or social novelty have been identified in two different progranulin (Grn)-deficient models (41,42). Furthermore, alternative social interaction paradigms, such as the resident-intruder test, are impaired in Grn mutant mice (43,44). Interestingly, reducing inflammation in a Grn mouse model can rescue social novelty defects (45) indicating that the microgliosis we observe in mutant CHMP2B mice may contribute to the social novelty phenotype. Such social behaviours in mice can be influenced by other physiological and environmental factors; importantly, here we did not observe any other behavioural changes in a range of additional tests including those for olfaction, novelty-induced locomotor activity, spatial novelty preference or depression-like behaviour. This indicates a very specific defect in social behaviour occurs in our CHMP2B^Intron5^ model.

Furthermore, our CHMP2B^Intron5^ mice do not show behaviours indicative of anxiety. In contrast, other FTD-relevant mouse studies report non-anxious phenotypes in approach-avoidance paradigms, often cited as an indicator of disinhibition-like behaviour. These findings are not universal, however. Increased time in the open arms of an elevated plus-maze is observed in certain *Grn-*deficient mutants (42), tau-encoding *MAPT* transgenics (46,47) as well as the CamKIIa-driven CHMP2B model (38). Yet a different *Grn-*deficient mutant (43) and the recently described PLB_tau_ model – a mutant that expresses a single copy of human FTD-associated mutant tau – both show heightened anxiety (48). Therefore, data from these types of behavioural paradigms in the mouse needs to be interpreted with care and must be considered in context of the other behavioural and neuropathological observations (40).

Finally, we also observed deficits in grip strength and motor co-ordination at 18 months of age, indicating late-stage motor deficits. These findings are consistent with motor-deficits in a recently reported transgenic CHMP2B^Intron5^ mouse model that drives expression using the neuronal Thy1.2 promoter (49), as well as the presence of motor symptoms at late stages of disease in patients with *CHMP2B*-FTD (29). Importantly, these motor problems did not confound interpretation of the social novelty data, as the distance travelled by CHMP2B^Intron5^ mice during testing was unaffected. Furthermore, we observed equivalent activity of CHMP2B^Intron5^ mice compared with control groups in other apparatus (e.g. elevated plus maze, Y-maze and T-maze) at 18 months of age. A slight reduction in activity was noted in the open field (Supplementary Material, Fig. S2B), likely a reflection of the motor dysfunction, although this was not observed until 40 min into the test; notably, this is beyond the exposure time in the apparatus used for the other behavioural paradigms so would not affect these results. The motor changes were accompanied by marked microgliosis in the spinal cord and altered muscle fibre type composition at 18 months of age. While not conclusive, these data are consistent with the distal denervation reported in the Thy1.2 CHMP2B transgenic model (49).

### Microglial phenotypes in *CHMP2B* patient brain

We used RT-PCR to measure the expression levels of genes implicated in inflammation in the frontal cortex of *CHMP2B* patients and age-matched controls. Our results show a trend towards a proinflammatory profile, with a slight increase in microglial proliferation pathways and proinflammatory cytokines. However, no statistically significant changes were observed so this alone cannot be viewed as evidence of a microglial contribution for *CHMP2B*-FTD. Confounds such as varying post-mortem delays and degrees of neuronal loss across samples also make such data hard to interpret. We therefore also investigated microglia in *CHMP2B* patient brain using immunohistochemistry. We identified microglia with striking resemblance to those observed in late-stage 18-month-old mutant *CHMP2B* mouse brain. In both cases, the microglia had an enlarged activated appearance and were full of the dense lysosomal-storage type aggregates we recently reported to be a hallmark *CHMP2B* neuronal pathology (15). Intriguingly, the first in-depth neuropathological description of *CHMP2B*-FTD (prior to the identification of the *CHMP2B* mutation) noted the presence of abnormal diffuse ubiquitin staining in microglia (29), which may represent the pathology we observe here. This convergence of end-stage microglial pathology in *CHMP2B* patient brain and our CHMP2B^Intron5^ mouse model indicates that the earlier microglial activation we observe in the mice may be relevant to disease pathogenesis.

### Microglial dysfunction in FTD

There are interesting parallels between FTD caused by *CHMP2B* and *GRN* mutation. Both are characterized by lysosomal storage pathology (15,50). We now report early microglial activation in CHMP2B mutant mice that precedes neuronal loss and behavioural changes. Similarly, microglia have been implicated in FTD caused by *GRN* mutation. Microgliosis accompanied by behavioural changes occurs in several different *Grn* homozygous knockout (*Grn -/-*) mouse models (41,42,44,51). Reducing aberrant microglial activity reduces behavioural changes (52,45), suggesting microglia play an important role in neurodegeneration caused by progranulin deficiency. However, it is important to differentiate between heterozygous (*Grn +/-)* and homozygous (*Grn -/-*) knockout mice, as dosage-dependent effects cause distinct pathologies in human patients. Individuals homozygous for *GRN* mutations present with neuronal ceroid lipofuscinosis (NCL), a form of lysosomal storage pathology, whilst heterozygous *Grn* mutations result in FTD (17,53). Interestingly, a comparison of *Grn *+/- mice to *Grn -/-* mice, found no gliosis in the heterozygous *Grn* state at 12 months, although the heterozygous animals do show sociability defects from 6 to 7 months of age (41). Thus in this *Grn* +/- mouse model overt microgliosis does not appear to play a role in producing at least some FTD-like behavioural changes.

Reducing microglial activation has also been reported to reduce tau pathology and neurodegeneration in the rTg4510 transgenic tau model of FTD (54). In addition, *C9orf72* knockout mice have impaired macrophage and microglial function characterized by a proinflammatory phenotype (55). While loss of C9orf72 is not generally considered the major driver of disease (56), loss of function and microglial alterations could well contribute to disease progression. In addition, mutations in the microglial gene *TREM2* have been identified in FTD cases (57) and cause altered microglial function (58). Therefore, there is evidence that microglia may play a role in several different genetic forms of FTD.

Neuroinflammation has been implicated in Alzheimer’s disease, Parkinson’s disease and amyotrophic lateral sclerosis (18,20). Recent genetic association studies performed in Alzheimer’s disease suggest that neuroinflammation is not simply a consequence of emerging pathological events associated with degeneration, but instead a driver of pathology (59,60). Our observation of an early neuroinflammatory component demonstrated by increased numbers of microglial cells prior to behavioural signs or neuronal loss, points to a potential role of neuroinflammation in driving *CHMP2B* pathology. Strategies aimed at inhibiting microglial proliferation produce beneficial effects in mouse models of neurodegenerative diseases (20,22,24). Taken all together, the modulation of neuroinflammation emerges as a potential candidate for tackling FTD.

## Materials and Methods

### Mice

The mutant CHMP2B^Intron5^ expressing mouse line Tg153 was used and maintained as a homozygous line as previously described (15,25). Maintenance as a homozygous line precludes comparison to non-transgenic littermate controls. Therefore, Tg153 mice were compared with the full length human CHMP2B^Wildtype^ mouse line Tg168, which was maintained as a hemizygous line and Tg168 non-transgenic littermates, as previously described (15,25). Tg153 and Tg168 lines were backcrossed to C57BL/6J for 10 generations to ensure matched genetic background. Tg153 mice were then interbred for either one or two generations prior to initiating behavioural experiments.

### Cases

Brain specimens (described in Supplementary Material, Table S1) were obtained from the Queen Square Brain Bank for Neurological Disorders, UCL Institute of Neurology and the Frontotemporal Dementia Research in Jutland Association. The study was approved by the UCL Institute of Neurology and National Hospital for Neurology and Neurosurgery Local Research Ethics Committee.

### Immunohistochemistry

Mouse brains and spinal cords were immersion fixed in 10% buffered formal-saline, embedded in paraffin wax and sections cut at 3–4 μm thickness. Immunohistochemistry was previously described (15). Immunohistochemistry was performed as previously described (15,20). Sections were processed for dewaxing, and antigen retrieval was performed with boiling citrate buffer (pH 6.0) for 20 min. Sections were treated with 1% methanol + 30% H_2_O_2_ to block for endogenous peroxidase activity (only for bright field immunohistochemistry), and with 5% normal serum + 0.1% BSA for nonspecific binding. After rinses with PBS-0.1% Tween 20 (PBST), sections were incubated overnight at 4 °C with rabbit anti-Iba1 (Wako, 019-19741) and mouse anti-NeuN (Millipore, MAB377). For immunofluorescence, sections were incubated in Sudan Black in 70% EtOH for 10 min in order to reduce autofluorescence of the tissue. After washes with PBST, sections were incubated with the appropriated biotinylated (Vector Labs) or Alexa 488- and 594-conjugated secondary antibodies (Invitrogen). For bright field immunohistochemistry, following rinses sections were incubated with Vectastain ABC complex (Vectors Labs) and visualized with diamino-benzidine (DAB) precipitation. Sections were mounted with DePeX and imaged in a Leica DM5000B microscope, coupled to a Leica DFC300FX camera. For immunofluorescence labelling, nuclei were counterstained with DAPI and mounted with Mowiol/DABCO (Sigma-Aldrich) mixture. Sections were visualized on a Leica TCS-SP5 confocal system, coupled to a Leica CTR6500 camera. Quantification of microglial cells (*n*  =  3 per group) was performed from the cortex, thalamus and CA1 of the hippocampus, and represented as cells per square millimetre. Visual inspection of 3 mice of each genotype was used to identify DAPI condensation.

For Figure 7 only, Iba1 and β-III tubulin staining were performed as previously described (15) on three 18-month-old mouse brains and the frontal cortex of two *CHMP2B*-FTD patient brains (Iba1 staining only). Alexa Fluor conjugated secondary antibodies (Life Technologies) were used to visualize the staining. Images were collected using a 63× lens with a 1.4 NA on a Zeiss LSM 510 confocal microscope; autofluorescence was identified by its presence in all channels.

### RT-PCR

Mouse cortex, thalamus and hippocampus were dissected from female non-transgenic, CHMP2B^Wildtype^ and CHMP2B^Intron5^ brains under a microscope. Samples were homogenized in Trizol reagent (Invitrogen), following the manufacturer instructions to isolate RNA, as previously described (20). The isolated RNA was quantified (Nanodrop, Thermo Scientific) and retrotranscribed with iScirpt^TM^ cDNA Synthesis Kit (Biorad). cDNA libraries were analysed by RT-PCR using iTaq^TM^ Universal SYBR® Green Supermix (Biorad) for the following genes (Sigma-Aldrich): *Csf1* (NM_007778.4; forward (FW), agtattgccaaggaggtgtcag; reverse (RV), atctggcatgaagtctccattt), *Csf1r* (NM_001037859.2; FW, gcagtaccaccatccacttgta; RV, gtgagacactgtccttcagtgc), *Il34* (NM_00113 5100.1; FW, ctttgggaaacgagaatttggaga; RV, gcaatcctgtagttgatggggaag), P*u.1* (NM_011355.1; FW, cagaagggcaaccgcaagaa; RV, gccgctgaactggtaggtga), *Cebpa* (NM_ 007678.3; FW, agcttacaacaggccaggtttc; RV, cggctggcgacatacagtac), *Tgfb* (NM_011577; FW: tgtacggcagtggctgaacc, RV: cgtttggggctgatcccgtt), *Il1b* (NM_00836 1.3; FW, cagacccaccctgca; RV, accgtttttccatcttcttct), *Tnfa* (NM_013 693; FW: aggcactcccccaaaagatg, RV: ttgctacgacgtgggctac), *Irf8* (NM_008320; FW: cggggctgatctgggaaaat, RV: cacagcgtaacctcg tcttc), I*l6* (NM_031168.1; FW, tccagaaaccgctatgaagttc; RV, caccagcatcagtcccaaga), C*cl2* (NM_011333.3; FW: ttaaaaacctggatcggaaccaa, RV: gcattagcttcagatttacgggt), A*rg1* (NM_007482.3; FW, agcact ga ggaaagctggtc; RV, cagaccgtgggttcttcaca), *Runx1* (NM_001111021; RW: caggcaggacgaatcacact, RV: ctcgtgctggcatctctcat), *Il4* (NM_021 2 83 .2; FW, cctcacagcaacgaagaaca; RV, cgaaaagcccgaaagagtc) and *Gapdh* (NM_008084.2; FW, tgaacgggaagctcactgg, RV, tccaccaccctgttgctgta). Data were analysed by ΔΔCt method, using *gapdh* as a housekeeping gene.

Human frontal cortex RNA was extracted using QIAzol (Qiagen) from *CHMP2B*-FTD (*n* = 4) and age-matched controls (*n*= 5). Further details for each case are provided in Supplementary Material, Table S1. RNA was retrotranscribed as described above. cDNA libraries were analysed by RT-PCR using iTaq^TM^ Universal SYBR^®^ Green Supermix for the following genes (Sigma-Aldrich): *CSF1* (NM_000757.5; FW, agccacatgattgggagtgg; RV, tggatctttcaactgttcctggt), *CSF1R* (NM_005211.3; FW, cctgggacccttttctgacc; RV, aggtgtgcctgtatgtgtcc), *Il1B* (NM_000576.2; FW, ggctgctctgggattctcttc; RV, agtcatcctcattgccactgta), *TNFA* (NM_000594.3: FW, tccccagggacctctctcta; RV, gagggtttgctacaacatggg), *TGFB* (NM_000660.5; FW, ggaaattgagggctttcgcc; RV, cggtagtgaacccgttgatgt), *IL6* (NM_000600.4; FW, ggcactggcagaaaacaacc; RV, accaggcaagtctcctcattg), *IL10* (NM_000572.2; FW, cgagatgccttcagcagagt; RV, ggcaacccaggtaaccctta), *IL34* (NM_152456.2; FW, ttgtccctcttgaatgcccc; RV, gacggagctttgtttacagca), *IL4* (NM_000589.3; FW, ctttgctgcctccaagaacac; RV, ccaacgtactctggttggct), *ARG1* (NM_001244438.1; FW, aggaaagattcccgatgtgcc; RV, gtccacgt ctctcaagccaa), *RUNX1* (NM_001754.4; FW, ggtttcgcagcgtggtaaaa; RV, gcactgtgggtacgaaggaa), *IRF8* (NM_002163.2; FW, atcaaaaggagcccttcccc, RV, gggagaatgctgaatggtgc), *CEBPA* (NM_004364.4; FW, tataggctgggcttcccctt; RV, agctttctggtgtgactcgg), *CCL2* (NM_002982.3; FW, tcccaaagaagctgtgatcttca; RV, tttgcttgtccaggtggtcc) and gapdh (NM_002046.5; FW, tcggagtcaacggatttggt; RV, ttcccgttctcagccttgac).

### Protein analysis

Mouse cortical samples from wild type, CHMP2B^Wildtype^ and CHMP2B^Intron5^ brains were homogenized in RIPA buffer (Thermo Fisher) with protease inhibitors (EASYpack, Roche) and phosphatase inhibitors (PhosSTOP, Roche). Protein was quantified using BCA assay (Thermo Fisher) following the manufacturer’s instructions. Assessment of inflammatory cytokines was performed using the V-PLEX Plus Proinflammatory Panel 1 Kit (Meso Scale Discovery).

### Magnetic resonance imaging (MRI)

Microscopic MRI was performed as previously described (26) on female mice: 10 CHMP2B^Intron5^, 7 CHMP2B^Wildtype^ and 10 non-transgenic controls. One CHMP2B^Intron5^ mouse was excluded from the analysis due to the presence of a brain tumour identified by the MRI. Briefly, mice were perfusion-fixed with 10% buffered formal-saline doped with 8 mM Gd-DTPA. After this, mice were decapitated and skin, muscle, lower jaw, tongue, nasal bones and zygomatic arches removed, with the remaining intact skulls post-fixed in 10% buffered formal-saline containing 8 mM Gd-DTPA at 4 °C for 9 weeks before imaging. All imaging was performed with a 9.4T VNMRS horizontal bore scanner (Agilent Inc.). An imaging gradient set with a 60 mm inner diameter (SGRAD 115/60/HD/S, Agilent Technologies UK Ltd., Berkshire, UK) was used. A 35L mm birdcage RF coil was employed for RF transmission and signal detection. Tuning and matching of the coil was performed manually. Prior to imaging, samples were removed from fixative and excess solution carefully blotted with a paper towel. They were then immersed in Fomblin perfluoropolyether (type PFS-1, Solvay Solexis S.p.A., Bollate, Italy) in 50 ml plastic syringes (permitting three samples to be imaged simultaneously) and immobilized with surgical gauze. Samples were allowed to equilibrate for at least 2 h at room temperature prior to imaging. Air bubbles in samples were minimized by the equilibration time and gentle agitation of the syringe. High resolution *ex vivo* structural images were acquired using a three-dimensional (3D) gradient echo sequence with the following parameters: Field-of-view  =  32 mm  ×  25 mm  ×  25 mm; resolution = 40 μm  ×  40 μm  ×  40 μm; repetition time = 17 ms; echo time = 4.54 ms; flip angle = 51°; averages = 6. Total imaging time was 11 h 36 min. Up to three samples were acquired at a time.

### MRI image analysis

Brain images were manually masked and cropped from multiple-subject scans using ITK-Snap software (61) and oriented to the same standard space. N3 bias correction was applied to correct image intensity non-uniformities (62). Brain tissue was automatically separated from skull using a multi-atlas parcellation approach, Similarity and Truth Estimation for Propagated Segmentations (63,64), employing an external mouse brain atlas database (65) and open-source registration and segmentation software, *NiftyReg* and *NiftySeg* (available: http://cmictig.cs.ucl.ac.uk/research/software). To align equivalent anatomical regions between subjects, we performed group-wise registration of all brain images, using *NiftyReg* and 1 iteration rigid registration, 4 iterations affine registration, and 20 iterations non-rigid registration (66), with normalized mutual information used as the similarity measure. Finally, we performed Tensor-Based Morphometry (67), using the deformation fields generated during registration. We found the Jacobian determinant at each voxel, log-transformed these values, and smoothed with a 3D Gaussian kernel (FWHM 0.16mm). We then used the general linear model to compare local volume differences between groups, with brain volume, from the brain masks, used as a covariate.

### Stereology tissue processing

Five CHMP2B^Intron5^ and five non-transgenic brains were cut horizontally in 60 µm sections and all sections were mounted on Superfrost plus object glasses (Thermo Scientific) in the order of cutting. After drying the sections were stained with KaryoMAX Giemsa Stain Solution (Life Technologies) for 20 min, destained in 1% acetic acid for 30 min, dehydrated in series of baths of ethanol and xylene and cover slipped with DPX-mounting medium.

### Defining the region of interest for stereology

We used the MRI scans to identify the region of interest [the thalamus was chosen as it had highly significant volume loss, and is known to be affected in FTD (35)], and the mouse brain atlas (Franklin and Paxinos, 3rd edition, 2007) to pinpoint specific anatomical structures for delimiting the counting area. The region of interest was delimited at the front by the ventral hippocampal commissure, capsula interna and the reticular nucleus, and at the back by a line going from the ‘anterior end’ of the dentate gyrus to the midline. In the more ventral sections, this line went in front of the Lithiod nucleus (when it became visible).

### Stereological cell counting

The total number of neurons in the thalamus was estimated using an unbiased stereological counting method as previously described by West and colleagues (68) by a researcher blind to mouse genotype. Briefly, using an Olympus BH2 microscope controlled by CAST software (Olympus) the region of interest in each section was marked at low magnification (2× objective). Subsequently, neurons were counted using the 100× objective by stepping through the region of interest with predetermined steps in the x and y directions (300 µm in both directions) in a raster pattern. Due to shrinkage of the tissue after drying and dehydrating, the average tissue thickness was 12.67 µm, and we therefore chose a dissector height of 8 µm, a counting frame size of 240 µm^2^ and a section sampling fraction of 3 to obtain a sampling volume, at which we counted between 100 and 200 neurons in each animal. From these data, we calculated the total number of neurons in the thalamus of each animal. Neurons were distinguished from other cell types as described by Hou *et al.* (69).

### Behavioural testing

Behavioural testing was carried out on female mice using cohorts of 12 CHMP2B^Intron5^, 12 CHMP2B^Wildtype^ and 12 non-transgenic controls. Female mice were used in order to keep the cohort sex-matched and to reduce potential losses due to fighting. Mice were analysed in parallel at 6, 12 and 18 months of age in the order of tests as described below over an approximately 4-week period. One CHMP2B^Intron5^ mouse died prior to testing at 18 months of age. Mice were housed on a 12/12-h light/dark cycle (lights on at 0700 h and off at 1900 h). Mice were housed in groups of 3–4 with *ad libitum* food and water. All testing was performed during the light phase. To avoid habituation to the testing environment, different rooms were used for each paradigm at each timepoint. All mice were weighed at monthly intervals and no significant difference was observed between genotypes at any timepoint (Supplementary Material, Fig. S3E). All behavioural procedures were performed in accordance with the United Kingdom Animals (Scientific Procedures) Act of 1986 and the University of Oxford Policy on the Use of Animals in Scientific Research. All experiments were approved by the University of Oxford Animal Welfare and Ethical Review Board.

### Locomotor activity

To measure novelty induced locomotor activity (LMA), experiments were conducted in the light phase between 1200 and 1400 h. Ambulations were measured in transparent Plexiglass cages (20 × 35 cm), equipped with infrared photobeams (San Diego Instruments) over a total of 2 h.

### Motor function

To test motor function, mice were placed on a grooved plastic beam of a commercial rotarod device (Ugo Basile), which revolves at 5 rpm, facing in an orientation opposite to the rotation. After 30 s, the speed of rotation was increased from 5 to 40 rpm over 5 min. The time latency to fall from the rod or complete two rotations on the rod without an attempt to run on the accelerating rod was recorded. Three single trials on consecutive days were carried out. For grip strength analysis, mice were placed on a wire mesh 40 cm^2^ and the grid was upturned so that the mouse was hanging upside-down. The time taken for the mouse to fall from the grid was recorded up to a maximum of 3 min.

### Sociability and preference for social novelty behaviour

Sociability and preference for social novelty behaviour were assessed based on a previously described method (70). The apparatus was a rectangular, three-chambered arena (left and right chambers 20 × 30 × 30 cm; centre chamber 20 × 20 × 30 cm; total size 60 × 30 × 30 cm). Dividing walls were made from clear acrylic, with small square openings (4 × 4 cm) allowing access from the centre chamber into left and right chambers. Each chamber was cleaned and fresh bedding added between trials. A video camera, mounted in front of the apparatus, recorded each session. The paradigm consisted of a three-stage procedure. Stage (1) habituation: in the initial stage, the test mouse was first placed in the centre chamber and allowed to explore all three chambers of the apparatus for 5 min. It was then replaced in the centre chamber for a further 5 min with access to the left and right chambers denied by Plexiglasss doors. Stage (2) sociability: an unfamiliar BALB/c mouse (Stranger 1: weight- and sex-matched) was placed in either the left or right chamber enclosed in a small, internal wire cage (10 cm diameter × 12 cm) which allowed nose contact but prevented fighting; placement of Stranger 1 in the left or right chamber alternated between trials, with an empty but otherwise identical wire cage in the opposite chamber. Each stranger mouse had been habituated to placement in the small wire cage 24 h before testing. Following placement of Stranger 1 into the left or right chamber, both doors to these side chambers were then opened and the test mouse was allowed to leave the centre chamber and explore all three chambers of the apparatus for 5 min. The test mouse could therefore distribute its behaviour between the centre chamber, the chamber containing Stranger 1 or the opposite, empty chamber. Time spent in each compartment was recorded, with entry into any chamber defined as all four paws in that chamber. Stage 3) Preference for social novelty: each test mouse was immediately returned to the centre chamber and the doors to the side chambers were closed. Then followed a second 5 min session to quantify social novelty preference toward a novel stranger. With the initial stranger (Stranger 1; now familiar) retained in its original chamber, a second, unfamiliar mouse (Stranger 2) was placed in the previously empty but otherwise identical small wire cage in the opposite chamber. Following placement of Stranger 2 into the chamber opposite to that still containing Stranger 1, both doors to the side chambers were opened and the test mouse was allowed to leave the centre chamber and explore all three chambers of the apparatus for a second period of 5 min; it could therefore distribute its behaviour between the centre chamber, the chamber containing the previously investigated and now familiar mouse (Stranger 1) and the opposite chamber containing the novel, unfamiliar mouse (Stranger 2). All other parameters and measures were as described above for Stage 2. All testing was counterbalanced for left or right positioning of the stranger mice.

### Olfactory Preference Test

As a measure of the sense of smell, olfactory response to both attractive and aversive scents was assessed as previously described (71).

### Statistical Analysis 

For immunohistochemistry, RT-PCR and protein analyses, data are expressed as mean ± SEM, and analysed using one-way ANOVA, applying Tukey *post hoc* when necessary with Prism 6 (GraphPad) software. For stereological cell counts a t-test was performed to compare cell numbers in CHMP2B^Intron5^ and non-transgenic thalamus using Prism 5 (GraphPad). For behavioural data, pairwise comparisons and interactions were performed using ANOVAs with additional post-hoc tests as stated. Analysis was carried out using Prism 5 (GraphPad) or SPSS v.20 (IBM).

### Supplementary Material

Supplementary Material is available at *HMG* online.

## Supplementary Material

Supplementary Data
