# Supplementary material for: Early microgliosis precedes neuronal loss and behavioural impairment in mice with a frontotemporal dementia-causing CHMP2B mutation
Source: Hum Mol Genet. 2017 Jan 16;26(5):873–87. doi: 10.1093/hmg/ddx003 (PMC5409096; doi:10.1093/hmg/ddx003)
Supplement: Supplementary Data [file ddx003_Supp.pdf]

Clayton, Mancuso et al

Early microgliosis precedes neuronal loss and behavioural impairment in mice with a frontotemporal dementia-causing CHMP2B mutation

Supplemental Material

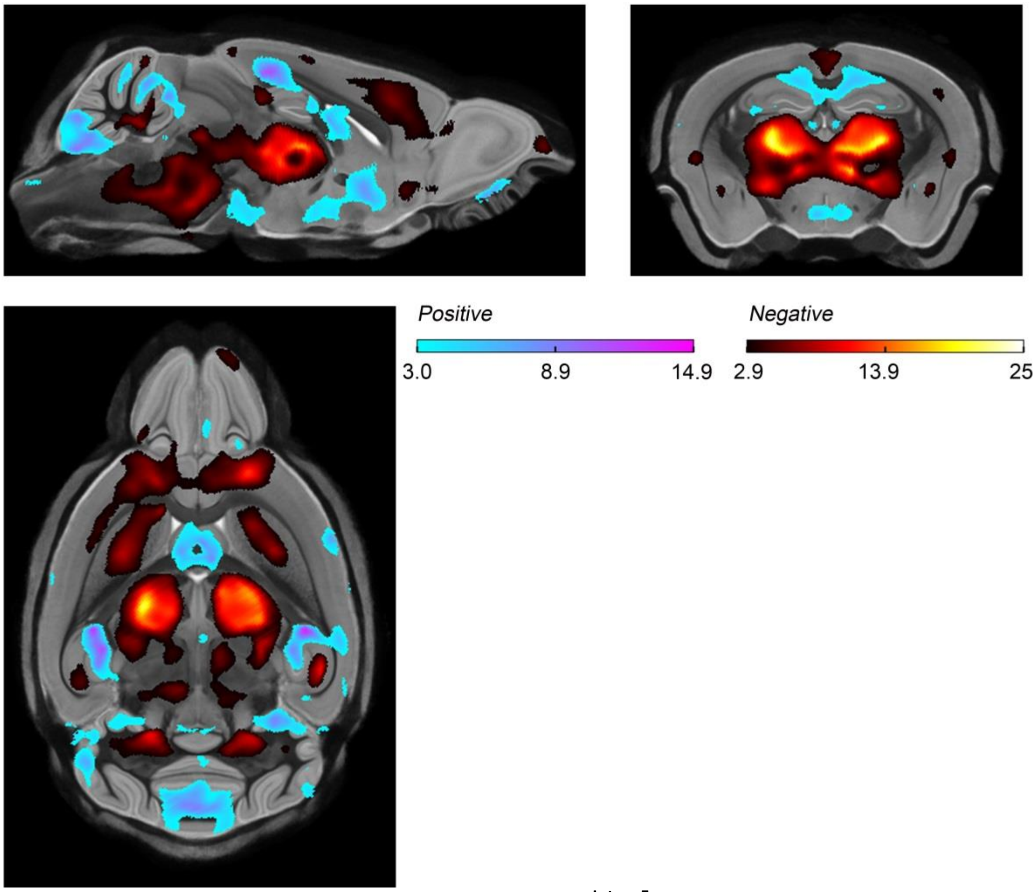

Figure S1. Volume loss in 18-month-old CHMP2B^{Intron5} mouse brain.
(A) High resolution *ex vivo* MRI results, showing statistically significant FDR-corrected (q=0.05) t-statistics overlaid on the group-wise registration average image, revealing local structural differences between the brains of the CHMP2B^{Intron5} mice (n=9) relative to CHMP2B^{Wildtype} mice (n=7). Regions highlighted in red represent a volume decrease in the CHMP2B^{Intron5} brains, and regions highlighted in blue represent a volume increase. Significant volume loss can be readily observed in the thalamus, cortex and brain stem of CHMP2B^{Intron5} brains. Sagittal, coronal and horizontal views are shown.

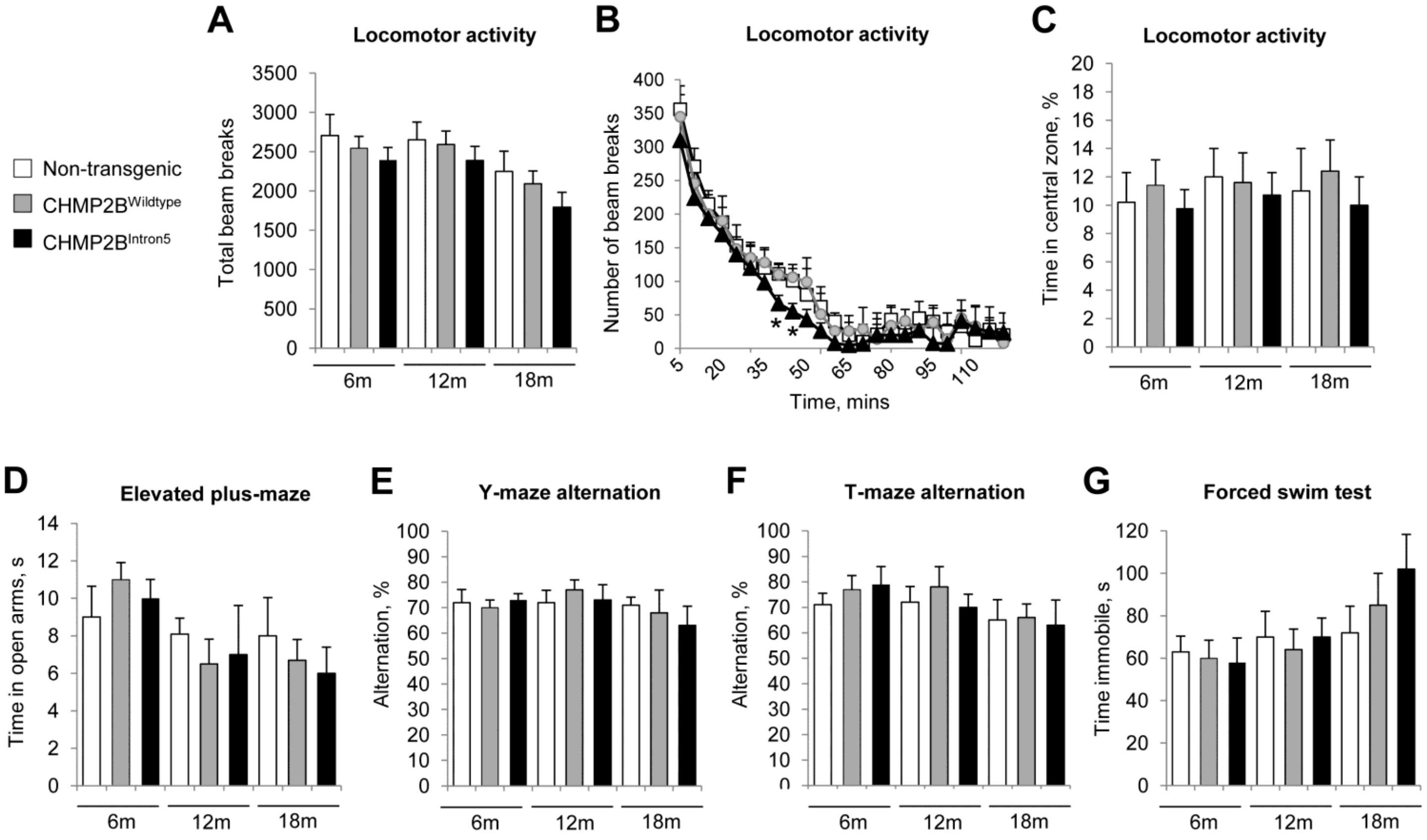

Figure S2. Longitudinal behavioural phenotyping of CHMP2B$^{\text{Intron5}}$, CHMP2B^{Wildtype} and non-transgenic mice.
(A) In the 2-hour spontaneous locomotor activity task, there was no difference in overall activity at any timepoint, however at 18 months of age, when the data are divided into 5 minute bins **(B)**, there was a main effect of time bin on beam breaks ($F_{23,460}$ = 22.45, p < 0.001), but no genotype-by-time bin interaction. CHMP2B$^{\text{Intron5}}$ mice were significantly less active between 40-50 minutes into the task compared to non-transgenic controls (* p < 0. 05; ANOVA with Bonferroni post-hoc test). **(C)** As measurements of anxiety-like behaviour, there was no difference in in the proportion of activity the central zone of the locomotor activity apparatus, or (**D**) time spent in the open arms in the elevated plus maze at any timepoint. In tests of short-term memory, both spatial novelty preference in the Y-maze **(E)** and spontaneous alternation in the T-maze **(F)** there was no difference in performance between genotypes. There was no difference in the distance travelled in any of the apparatus between genotypes during the tasks (D) and (E) (data not shown). **(G)** In the forced swim test for apathetic behaviour, there was also no difference in time spent immobile between genotypes. Data are shown as mean ± SEM.

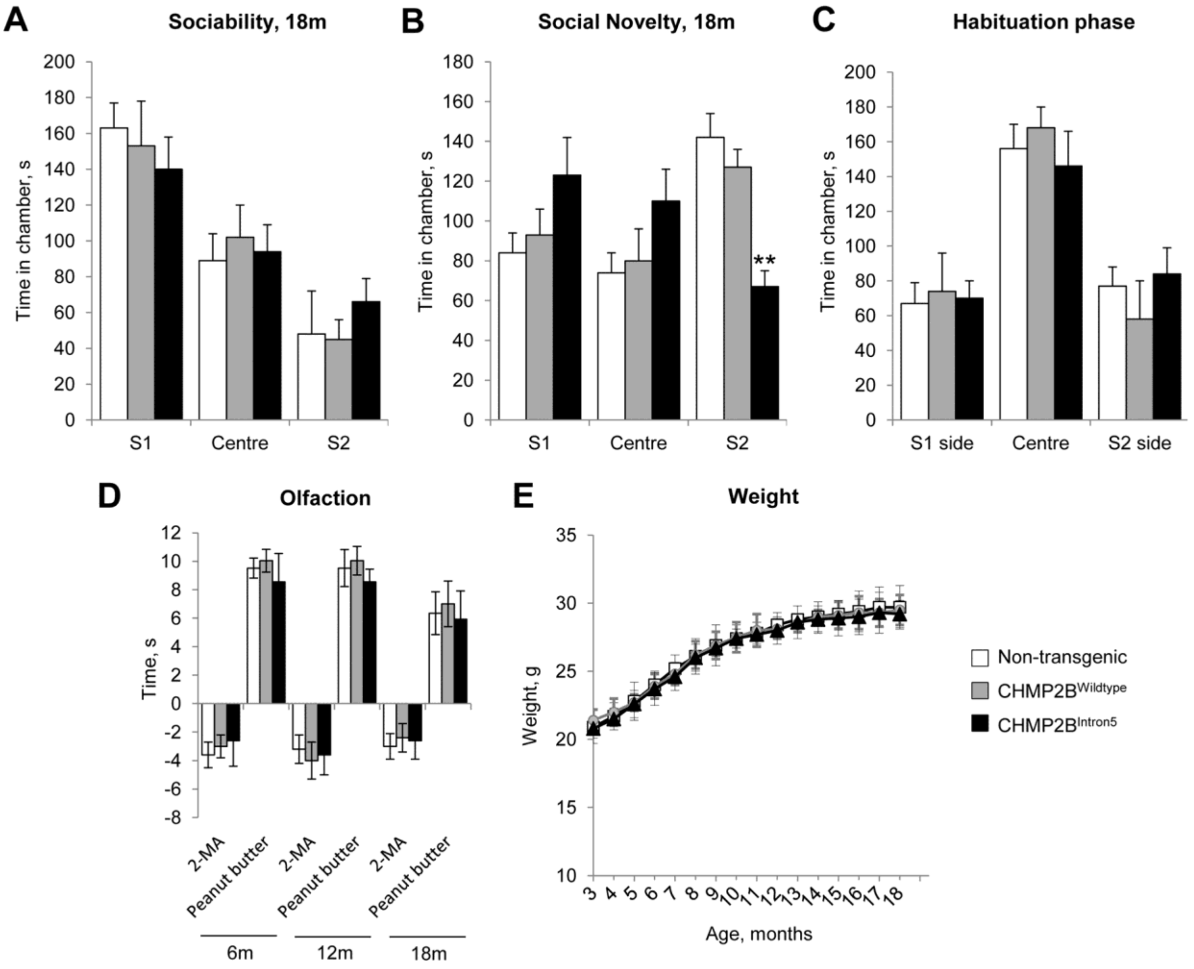

Figure S3. Further longitudinal behavioural phenotyping of CHMP2B^{Intron5}, CHMP2B^{Wildtype} and non-transgenic mice.

(**A**) In the first phase of the social interaction test at 18 months of age, there was no difference in the time spent in the chamber with stranger 1 (S1) between genotypes. In the social novelty phase of the task (**B**), CHMP2B^{Intron5} mice spent significantly less time with the new stranger 2 (S2) compared to non-transgenic controls (* $p < 0.01$; ANOVA with Bonferroni post-hoc test). (**C**) During the habituation phase of the same task, there was no difference in the average exposure time between the individual chamber sections between genotypes. **(D)** Olfactory performance was not altered between genotypes at any timepoint, with all animals spending more time with the attractive (peanut butter) versus the aversive (2-MA) odour. **(E)** Animals were weighed once a month and no significant differences were observed at any timepoint. Data are shown as mean ± SEM.

A

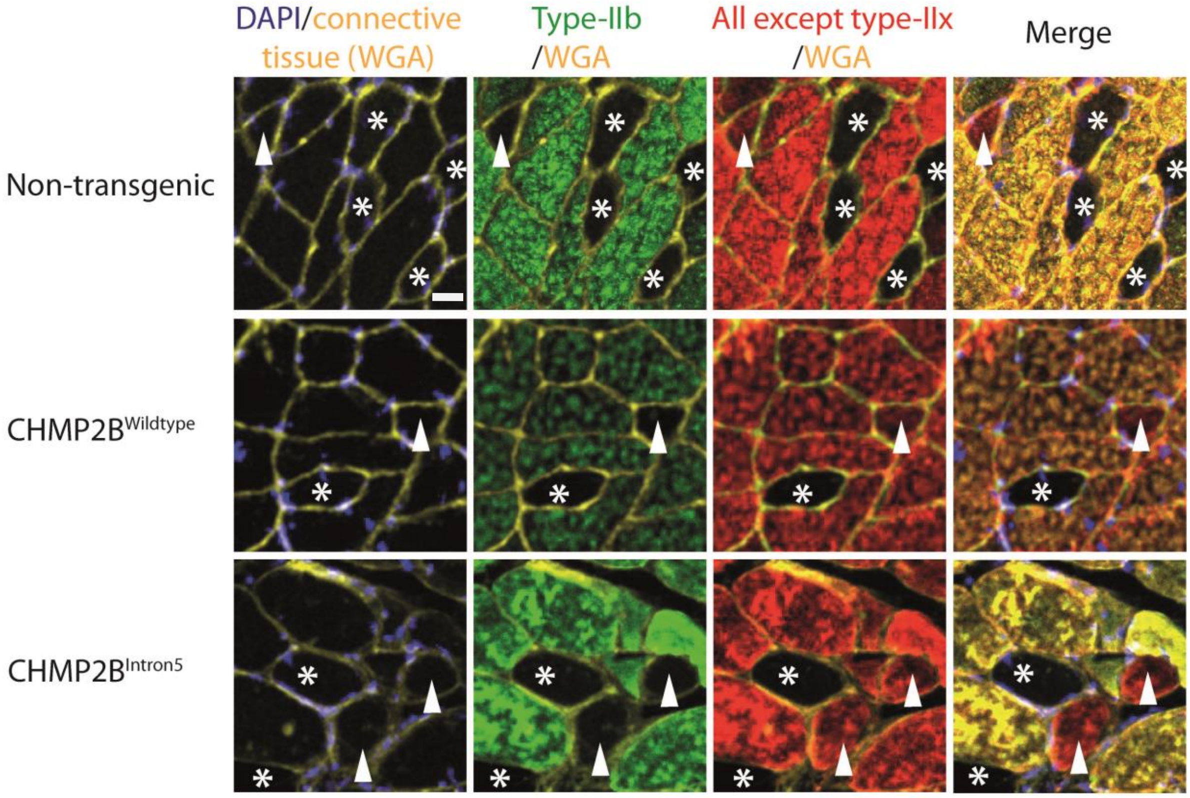

B

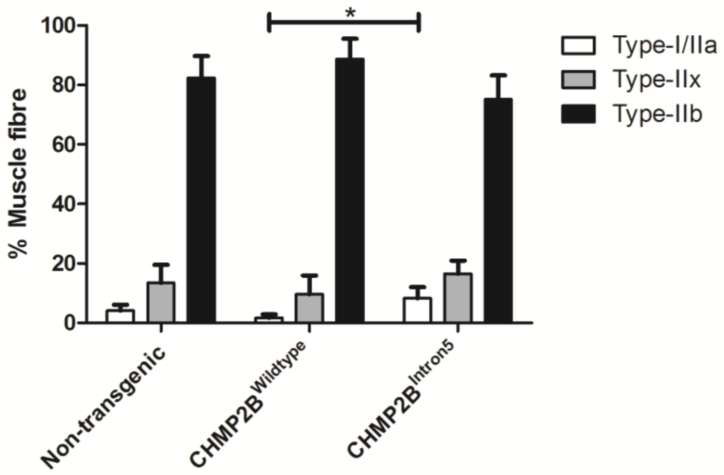

Figure S4. Quadriceps muscle fibre type composition in 18-month old non-transgenic, CHMP2B^{Wildtype} and CHMP2B^{Intron5} mice.

Representative confocal images **(A)** and quantification **(B)** of muscle fibre type percentage in quadriceps muscles of CHMP2B^{Intron5}, CHMP2B^{Wildtype} and non-transgenic mice (n=5, 3, 5). DAPI (blue) stains nuclei, MYH4 (green) recognizes Myosin Heavy Chain Type IIb and is used to identify type IIb fibres, MYH2 (red) recognizes more than one myosin isoform and is used to identify all fibre types except type IIx, Wheat Germ Agglutinin (WGA) (yellow) stains connective tissue and is used to outline muscle fibres. Arrowheads indicate type IIa fibres, asterisks indicate fibre type-IIx. Scale bar: 20µM. Error bars represent SEM, *p<0.05, ANOVA with Tukey post-hoc test.

Diagnosis	Sex	Age at death	PMI (hours)
FTD-3	F	61	NA
FTD-3	F	69	18
FTD-3	F	78	20
FTD-3	M	54	30
Control	F	64	79
Control	M	63	42
Control	M	72	13
Control	M	57	79
Control	M	71	39

Table S1. Human cases used for RNA analysis. PMI – post-mortem interval, NA – not available.

Supplemental Methods

Mouse Behaviour

Elevated Plus Maze (EPM)

The elevated plus maze (EPM) consisted of arms 30 cm long and 5 cm wide with the 2 opposing closed arms with 30 cm sides. Mice were placed in the EPM facing the open arm and allowed to freely explore the apparatus over 5-min trial. Entry into an arm of the EPM was defined using the base of the tail as the tracking position.

Anxiogenic Open Field

Behaviour was assessed in a white, anxiogenic open field (60 cm diameter, lighting at 3000 lux) Mice were placed into the apparatus at the edge and the distribution of time spent in the central (20 cm diameter) and peripheral areas was taken as a measure of anxiety. The total distance travelled was also recorded.

Spatial Novelty Preference in the Y-Maze

Spatial novelty preference was assessed in an enclosed Perspex Y-maze with arms of 30 × 8 × 20 cm placed into a room containing a variety of extramaze cues. Mice were assigned 2 arms (the "start" and the "other" arm) to which they were exposed during the first phase (the exposure phase), for 5 min. This selection of arms was counterbalanced with respect to the genotype. Timing of the 5-min period began only once the mouse had left the start arm. The mouse was then removed from the maze and returned to its homecage for a 1-min interval between the exposure and test phases. During the test phase, mice were allowed free access to all 3 arms. Mice were placed at the end of the start arm and allowed to explore all 3 arms for 2 min beginning once they had left the start arm. An entry into an arm was defined by a mouse placing all 4 paws inside an arm. Similarly, a mouse was considered to have left an arm if all 4 paws were placed outside the arm. The times that mice spent in each arm were recorded manually and a novelty preference (alternation) ratio was calculated for the time spent in arms [novel arm/(novel + other arm)].

Spontaneous Alternation in a T-maze

The apparatus consisted of a black T-shaped wooden maze made of arms, measuring 30 cm in length, 10 cm in width, and 29 cm in height. The goal arm entrances were provided with sliding guillotine doors. A central partition wall, extending 7 cm into the start arm, divided the choice point into 2 goal arms to prevent the mouse from receiving any sensory input from the non-visited arm. A

mouse was placed in the start arm of the T-maze and allowed to choose a goal arm. The mouse was then confined to the goal arm by sliding the guillotine door down. The arm entered (left or right) was recorded and the mouse was allowed to explore the goal arm for 30 s. The mouse was returned to the start arm, with the guillotine doors re-opened and the central partition removed, and allowed to explore again. The goal arm entered on the second run was recorded (left or right) and the mouse was then returned to its home cage. Each mouse was tested twice each day for 5 days (a total of 10 trials). The percentage of trials in which the mouse entered a different goal arm on the second run (i.e., alternated) was calculated.

Forced swim test

*E*ach mouse was placed into a transparent 4l glass beaker (45 cm high × 15 cm diameter) filled to a depth of 20 cm with water (maintained at 25°C) and remained there for 6 min. The duration of immobility, which was defined as floating in an upright position without additional activity other than that necessary for the animal to keep its head above water, was recorded during the last 4 min of the test period.

Muscle fibre typing

Quadriceps muscles were flash-frozen in precooled isopentane, and embedded in optimal cutting temperature (OCT) compound (Tissue-Tek, Sakura). Cross sections (20 µm thickness) were cut on a cryostat (Bright). Fibre-type-specific myosin isoforms were immunolabeled with a combination of the following monoclonal antibodies: clone BF-F3 (type-IIb), clone BF-35 (all fibers except type-IIx) both from Developmental Studies Hybridoma Bank, and immunofluorescently labelled using goat anti-mouse IgM-AlexaFluor488, goat anti-mouse IgG-AlexaFluor568, and Wheat Germ Agglutinin (WGA)-Alexa633 (from Invitrogen). Nuclei were labelled with DAPI.
